# Anti-CD80/86 antibodies inhibit inflammatory reaction and improve graft survival in a high-risk murine corneal transplantation rejection model

**DOI:** 10.1038/s41598-022-08949-9

**Published:** 2022-03-22

**Authors:** Jun Zhu, Takenori Inomata, Masahiro Nakamura, Keiichi Fujimoto, Yasutsugu Akasaki, Kenta Fujio, Ai Yanagawa, Koichiro Uchida, Jaemyoung Sung, Naoko Negishi, Ken Nagino, Yuichi Okumura, Maria Miura, Hurramhon Shokirova, Mizu Kuwahara, Kunihiko Hirosawa, Akie Midorikawa-Inomata, Atsuko Eguchi, Tianxiang Huang, Hideo Yagita, Sonoko Habu, Ko Okumura, Akira Murakami

**Affiliations:** 1 Department of Ophthalmology, Juntendo University Graduate School of Medicine, 3-1-3 Hongo, Bunkyo-ku, Tokyo, 113-0033 Japan; 2 Department of Ophthalmology, Subei People’s Hospital Affiliated to Yangzhou University, Yangzhou, Jiangsu China; 3 Department of Strategic Operating Room Management and Improvement, Juntendo University Graduate School of Medicine, Tokyo, Japan; 4 Department of Hospital Administration, Juntendo University Graduate School of Medicine, Tokyo, Japan; 5 Department of Digital Medicine, Juntendo University Graduate School of Medicine, Tokyo, Japan; 6 Precision Health, Department of Bioengineering, Graduate School of Engineering, The University of Tokyo, Tokyo, Japan; 7 Center for Immune Therapeutics and Diagnosis, Juntendo University, Tokyo, Japan; 8 Morsani College of Medicine, University of South Florida, Tampa, FL USA; 9 Atopy Research Center, Juntendo University Graduate School of Medicine, Tokyo, Japan; 10 Department of Indoor Environment Neurophysiological Research, Juntendo University Graduate School of Medicine, Tokyo, Japan; 11 Department of Immunology, Juntendo University Graduate School of Medicine, Tokyo, Japan

**Keywords:** Allotransplantation, Immunosuppression, Innate immunity

## Abstract

We investigated the effects of anti-CD80/86 antibodies in a murine high-risk corneal transplantation rejection model. A mixed lymphocyte reaction (MLR) assay was conducted with anti-CD80/86 antibodies. Inflammatory cytokine levels in the culture supernatant were measured using an enzyme-linked immunosorbent assay. Interferon (IFN)-γ-producing CD4^+^ T cell frequencies in the MLR were assessed using flow cytometry. In vivo, high-risk corneal allograft survival and IFN-γ-producing CD4^+^ T cell frequencies in corneal grafts were assessed with intraperitoneal injection of anti-CD80/86 antibodies compared to phosphate-buffered saline (PBS). RNA-sequencing was performed on corneal grafts 2 weeks post-transplantation. Anti-CD80/86 antibodies significantly decreased T-cell proliferation, IFN-γ^+^-producing CD4^+^ T cell frequencies, and IFN-γ, interleukin (IL)-1β, IL-2, IL-10, and tumor necrosis factor-α production in the MLR compared to PBS injection. Intraperitoneal injection of anti-CD80/86 antibodies significantly prolonged corneal graft survival and decreased IFN-γ^+^-producing CD4^+^ T cell frequencies compared to PBS injection. Gene set enrichment analysis showed that the gene sets mainly enriched in the control group were related to allograft rejection and inflammatory response compared to PBS injection. Anti-CD80/86 antibodies significantly prolonged corneal graft survival by inhibiting T-cell proliferation and inflammatory response.

## Introduction

Corneal transplantation has been performed in over 180,000 cases worldwide^1^ annually with a high success rate. Acute corneal rejection has decreased with the advent of immunosuppressive drugs such as steroids, cyclosporine, and tacrolimus; however, corneal rejection still occurs in 40–90% of recipients with neovascularization due to infection, autoimmune disease, or re-transplantation^2,3^. Immunosuppressive drugs are still associated with several problems such as severe adverse effects including opportunistic infections, drug toxicity, cataract, and glaucoma^4,5^. Therefore, it is clinically important to establish long-term immune tolerance in transplanted corneal grafts.

In corneal transplantation rejection, recipient’s immune system cells derived from blood vessels newly formed by corneal transplantation recognize the transplanted donor cornea, as a foreign body, and antigen presentation occurs in the cervical lymph nodes, causing naive T cells to differentiate into effector T cells and destroy the target graft cornea^6^. Therefore, the corneal transplantations performed on existing corneal neovascularization and lymphatic vessels bed have been recognized to have a higher inclination to rejection^7–9^. It is known that this immune sensitization process involves major histocompatibility complex allorecognition and costimulatory pathway by the interaction between activated T cells and antigen-presenting cells (APCs)^10,11^.

Antigen-specific T-cell activation plays a critical role in corneal allograft rejection^12^. The interaction between CD80 and CD86 on APCs and the co-stimulatory CD28 receptor on T cells is regarded as a crucial signaling pathway in T-cell activation^13^. Blocking the CD28-CD80/86 pathway prolongs allograft survival in murine models of pancreatic and cardiac transplantation^14–16^. Physiological inflammatory and immune responses are regulated by inhibitory interactions between CD80/86 and CD152 (cytotoxic T-lymphocyte antigen-4 [CTLA-4]). This interaction is important in the immune checkpoint pathway, which counteracts T-cell activation^17^. Kagaya et al.^18^ investigated the role of CD80^+^ and CD86^+^ cells in the cornea and cervical lymph nodes in mouse corneal transplant models using anti-CD80 and anti-CD86 monoclonal antibodies and found that CD80 and CD86 blockade confers immunosuppression. Although blocking the CD80/86 signaling pathway prevents allograft rejection in corneal transplantation models^19^, the effect of CD80/86-CD28 blockade in a pre-established immune rejection environment, especially in a corneal microenvironment with local inflammation, has not been fully investigated. In this study, we aimed to investigate the effect of anti-CD80/86 antibodies on allogeneic immune responses in an inflammatory allogeneic rejection model.

## Results

### Anti-CD80/86 antibodies suppressed CD4^+^ T cell proliferation

**Figure 1:**
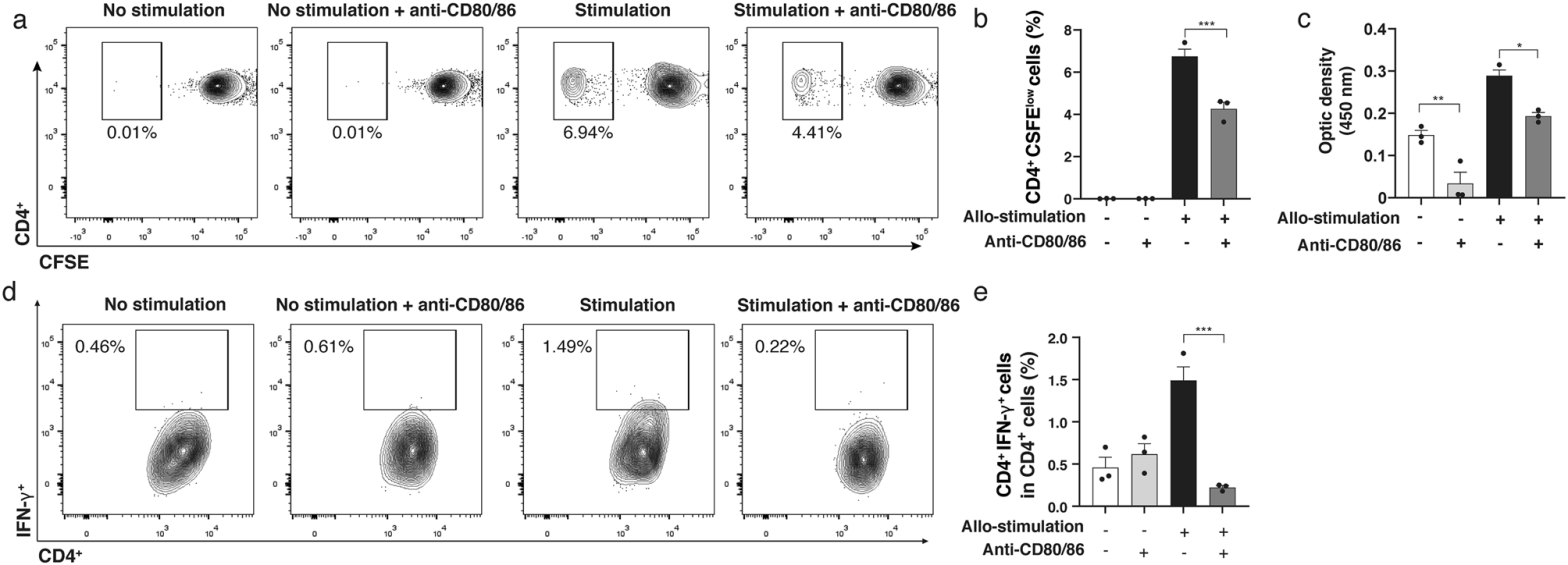
Anti-CD80/86 antibodies suppress T lymphocyte proliferation in mixed lymphocyte reaction assay. Representative flow cytometry plots of CD4^+^ (**a**) CFSE-labeled cells. (**b**) The frequency of CD4^+^ CFSE^low^ cells in CD4^+^ T cells was significantly reduced in the allogeneic stimulation group with anti-CD80/86 antibodies compared with that in without anti-CD80/86 antibodies group (n = 3, ***P < 0.001). (**c**) Representative columns of BrdU assay results. Anti-CD80/86 suppressed T cell proliferation compared to without anti-CD80/86 antibodies in allogeneic stimulation and no allogeneic stimulation (n = 3, **P = 0.007, *P = 0.019, respectively). (**d**) Representative flow cytometry plots of the frequency of IFN-γ^+^CD4^+^ T lymphocyte with or without allogeneic stimulation and anti-CD80/86 intervention. (**e**) Frequency of IFN-γ^+^CD4^+^ T lymphocyte in CD4^+^ T cells reduced following treatment with anti-CD80/86 antibodies compared with that without anti-CD80/86 antibody treatment in allogeneic stimulation (n = 3, * *P* < 0.001). Data are presented as mean ± SEM. *CFSE* carboxyfluorescein diacetate succinimidyl ester.

Lymphocyte proliferation in the mixed lymphocyte reaction (MLR) assay was significantly inhibited when anti-CD80/86 antibodies were administrated. Figure 1a shows the representative flow cytometry plots for CD4^+^ CFSE^low^ T cells in the MLR. On day 3 of incubation, the frequency of CD4^+^ CFSE^low^ T cells in the MLR was significantly reduced in allogeneic stimulation group with anti-CD80/86 antibodies compared with that in without anti-CD80/86 antibodies group (Fig. 1b; allogeneic stimulation group, 6.9% ± 0.3%; allogeneic stimulation with anti-CD80/86 antibodies group, 4.4% ± 0.3%; n = 3, *P* < 0.001). The BrdU assay showed a significant lower opacity density in the allogeneic stimulation with anti-CD80/86 antibodies group compared to without anti-CD80/86 antibodies in allogeneic stimulation and no allogeneic stimulation (Fig. 1c, n = 3;* P* = 0.007, *P* = 0.019, respectively).

### Anti-CD80/86 antibodies reduced interferon (IFN)-γ-producing CD4^+^ T lymphocytes in MLR

Figure 1d shows the representative flow cytometry plots for IFN-γ-producing CD4^+^ T cells in MLR. Allogeneic stimulation with anti-CD80/86 antibodies significantly decreased the frequency of IFN-γ-producing CD4^+^ cells compared with that without anti-CD80/86 antibodies (Fig. 1e; allogeneic stimulation without anti-CD80/86 antibodies group, 1.49% ± 0.16%; allogeneic stimulation with anti-CD80/86 antibodies group, 0.22% ± 0.02%; n = 3; *P* < 0.001).

### Anti-CD80/86 antibodies reduced IFN-γ, interleukin (IL)-1β, IL-2, IL-10, and tumor necrosis factor (TNF)-α production

**Figure 2:**
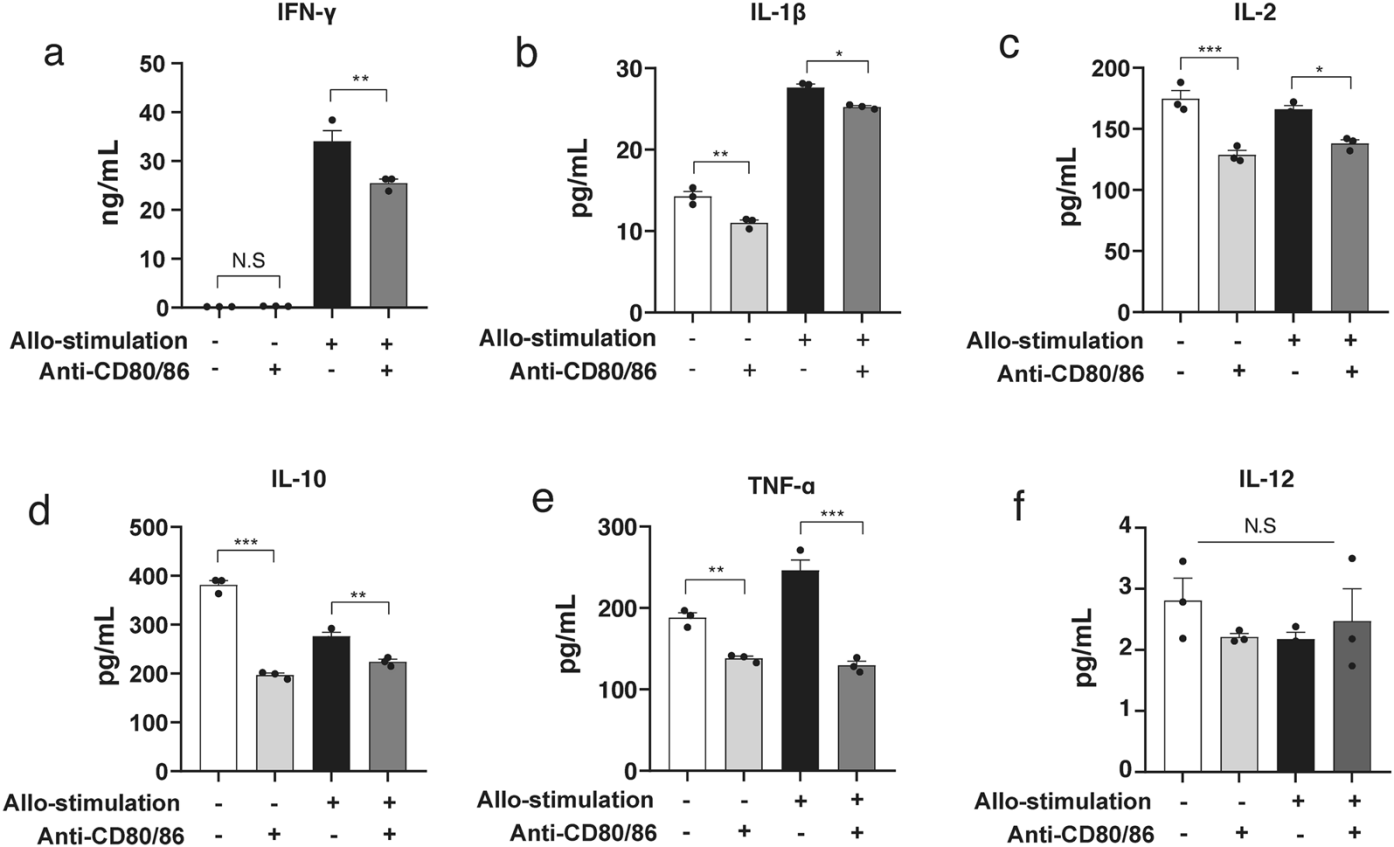
Anti-CD80/86 antibodies reduce pro-inflammatory cytokine production in mixed lymphocyte reaction. (**a**) IFN-γ production reduced by anti-CD80/86 antibodies compared with that without anti-CD80/86 antibodies (n = 3, **P = 0.005, N.S, *P* > 0.999, respectively). (**b**) IL-1β production reduced by anti-CD80/86 antibodies compared to that without anti-CD80/86 antibodies (n = 3, **P = 0.003, *P = 0.021, respectively). (**c**) IL-2 production reduced by anti-CD80/86 antibodies compared to that without anti-CD80/86 antibodies (n = 3, *P = 0.012, ***P < 0.001, respectively). (**d**) IL-10 production reduced by anti-CD80/86 antibodies compared with that without anti-CD80/86 antibodies (n = 3, **P = 0.004, ***P < 0.001, respectively). (**e**) TNF-α production reduced by anti-CD80/86 antibodies compared with that without anti-CD80/86 antibodies (n = 3, **P = 0.010, ***P < 0.001, respectively). (**f**) IL-12 production did not change between the groups (n = 3, *P* = 0.530).

Treatment with anti-CD80/86 antibodies significantly reduced the secretion of cytokines, including IFN-γ (Fig. 2a, allogeneic stimulation without anti-CD80/86 antibodies, 34.1 ± 2.2 ng/mL; allogeneic stimulation with anti-CD80/86 antibodies, 25.5 ± 0.8 ng/mL, n = 3; *P* = 0.005), IL-1β (Fig. 2b; allogeneic stimulation without anti-CD80/86 antibodies, 27.6 ± 0.4 pg/mL; allogeneic stimulation with anti-CD80/86 antibodies, 25.3 ± 0.2 pg/mL; n = 3; *P* = 0.021), IL-2 (Fig. 2c; allogeneic stimulation without anti-CD80/86 antibodies, 166.0 ± 3.1 pg/mL; allogeneic stimulation with anti-CD80/86 antibodies, 138.0 ± 3.1 pg/mL; n = 3; *P* = 0.012), IL-10 (Fig. 2d; allogeneic stimulation without anti-CD80/86 antibodies, 276.7 ± 7.7 pg/mL; allogeneic stimulation with anti-CD80/86 antibodies, 224.1 ± 5.2 pg/mL; n = 3; *P* = 0.004), and TNF-α (Fig. 2e; allogeneic stimulation without anti-CD80/86 antibodies, 246.2 ± 12.6 pg/mL; allogeneic stimulation with anti-CD80/86 antibodies, 129.5 ± 5.2 pg/mL; n = 3; *P* < 0.001). The secretion of IL-12 was no significant different between the groups (Fig. 2f; allogeneic stimulation without anti-CD80/86 antibodies, 2.2 ± 0.2 pg/mL; allogeneic stimulation with anti-CD80/86 antibodies, 2.5 ± 0.9 pg/mL; n = 3; *P* = 0.530).

### Anti-CD80/86 antibodies prolonged allograft survival

**Figure 3:**
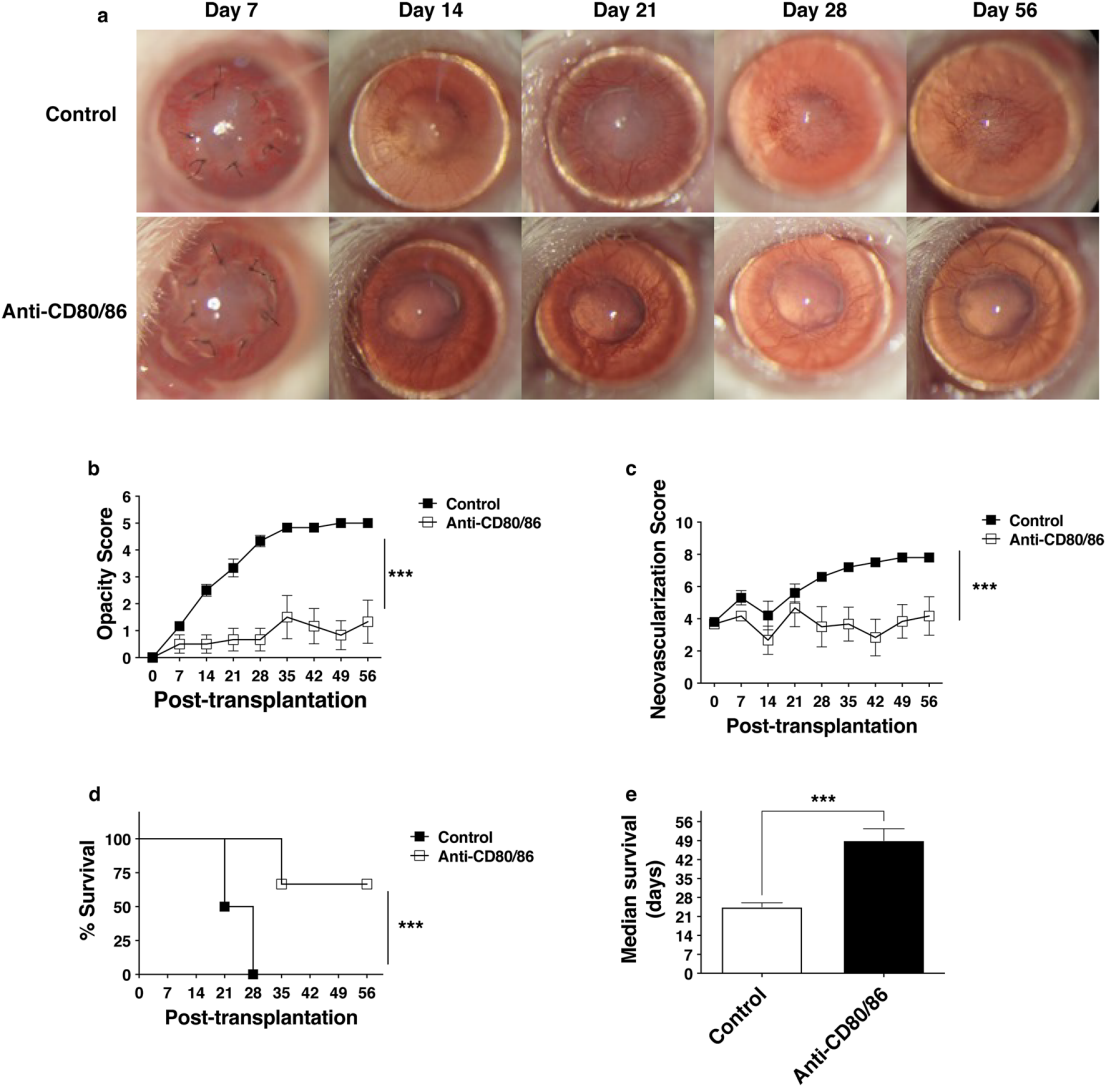
Anti-CD80/86 antibody injection prolongs corneal graft survival. (**a**) Representative slit-lamp microscopy showing grafted corneas on day 56 post corneal transplantation. (**b**) Graft opacity scores reduced significantly in grafts with anti-CD80/86 injection compared with that in the control group (two-way ANOVA, n = 6/group; ***P < 0.001). (**c**) The neovascularization score was considerably reduced in grafts with anti-CD80/86 injection compared with that in the control group (two-way ANOVA, n = 6/group; ***P < 0.001). (**d**) Kaplan–Meier survival curves show a significant increase in the survival of grafts with anti-CD80/86 injection compared with that in the control group (log-rank test, n = 6/group, ***P < 0.001). (**e**) The median graft survival shows that only grafts with anti-CD80/86 injection significantly improved graft survival in high-risk recipients to levels observed in the control group (unpaired *t*-test, n = 6/group. ***P < 0.001).

Corneal graft survival was compared between the phosphate-buffered saline (PBS) and anti-CD80/86 antibody injection groups (Fig. 3a). The opacity score was significantly lower in the anti-CD80/86 injection group than in the PBS injection group (Fig. 3b; anti-CD80/86 injection vs. control, n = 6, *P* < 0.001). The neovascularization score was significantly lower in the anti-CD80/86 injection group than in the PBS injection group (Fig. 3c, anti-CD80/86 injection vs. control, n = 6, *P* < 0.001). The survival rate was significantly higher in the anti-CD80/86 injection group than in the PBS injection group (Fig. 3d; anti-CD80/86 injection vs. control, n = 6, *P* < 0.001). The median graft survival data showed that the injection of anti-CD80/86 antibodies significantly improved graft survival in high-risk recipients (Fig. 3e; unpaired *t*-test, n = 6/group; anti-CD80/86 injection vs control: 56 [35–56] days vs. 24.5 [21–28] days, ***P < 0.001). Anti-CD80/86 antibody injection in vivo did not affect recipient survival and corneal epithelial wound healing (Supplementary Fig. S1).

### Anti-CD80/86 antibodies reduced IFN-γ-producing CD4^+^ T cells in the corneal grafts

**Figure 4:**
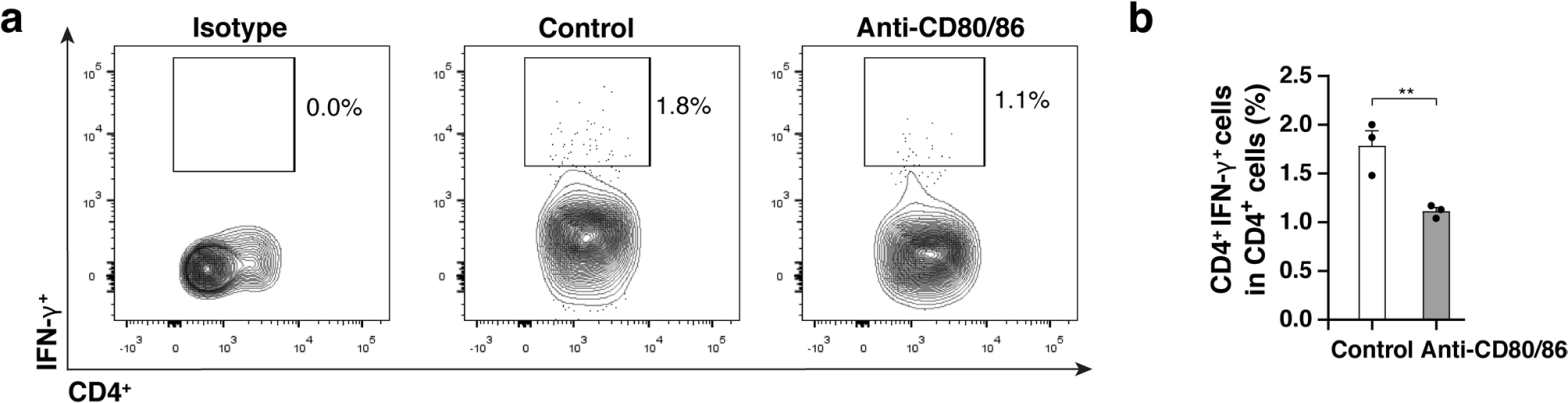
Anti-CD80/86 antibodies suppress IFN-γ-producing CD4^+^ T cells in corneal graft. (**a**) Representative flow cytometry plots of IFN-γ^+^CD4^+^ T cell frequency in the groups injected with anti-CD80/86 injection and phosphate-buffered saline (PBS). (**b**) Frequency of IFN-γ^+^CD4^+^ T cells in CD4^+^ T cells in the grafts of anti-CD80/86 injection mouse was lower than that in the grafts of PBS injection (n = 3, **P = 0.007). Data are presented as mean ± SEM.

Figure 4a shows the representative flow cytometry plots for IFN-γ-producing CD4^+^ T cells in the corneal grafts. Frequency of IFN-γ^+^CD4^+^ T cells in CD4^+^ T cells in the grafts of anti-CD80/86 injection mouse was lower than that in the grafts of PBS injection (Fig. 4b; anti-CD80/86 injection group, 1.1 ± 0.1%; PBS injection group, 1.8 ± 0.2%; n = 3; *P* = 0.007). Anti-CD80/86 antibody injection in vivo had no effect on the ratio of CD4^+^ T cells in total draining lymph nodes cells (Supplementary Fig. S2).

### Anti-CD80/86 antibodies downregulated the mRNA expression of pro-inflammatory cytokines

**Figure 5:**
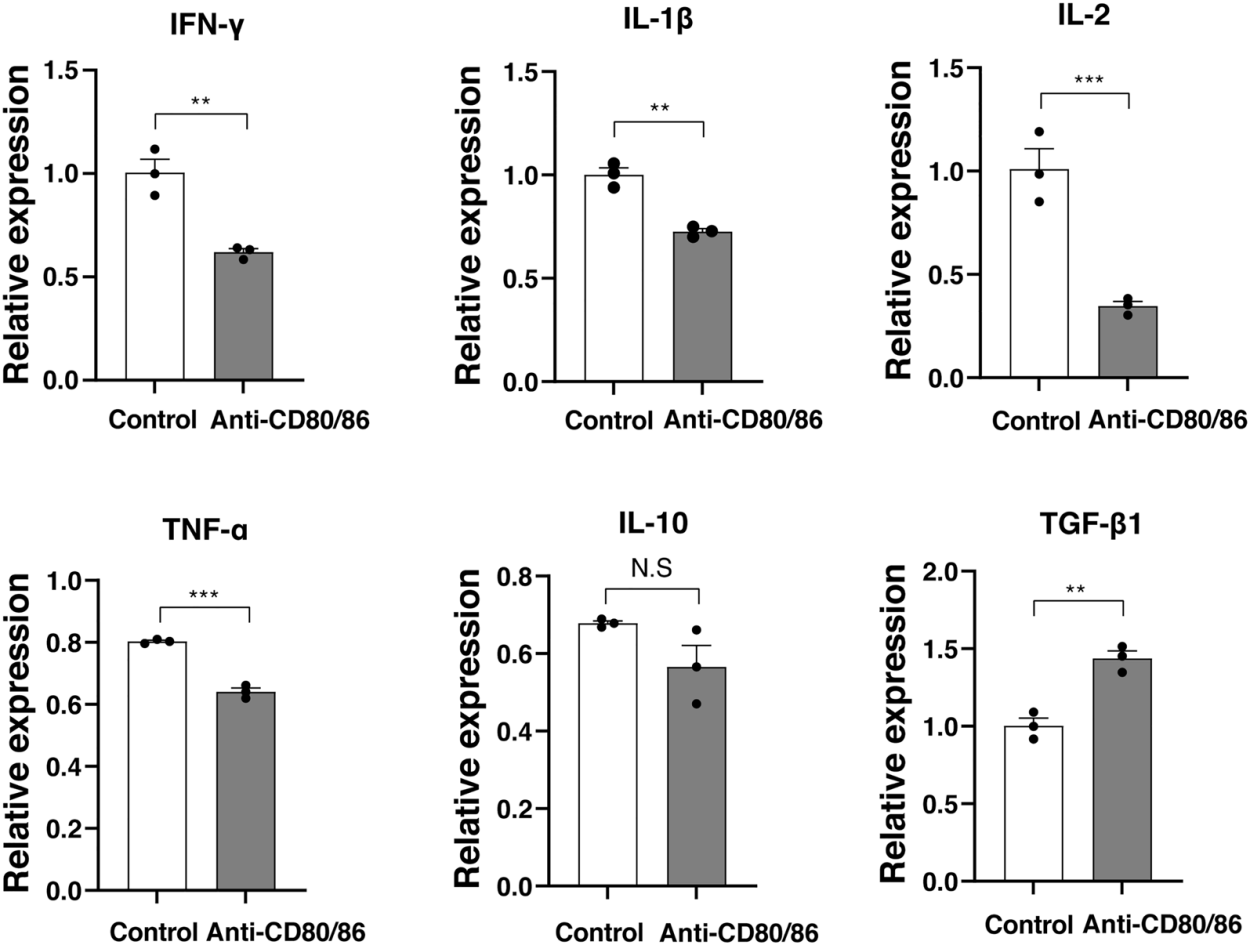
Anti-CD80/86 antibodies regulate cytokine expression in the corneal grafts. The relative mRNA expression of IFN-γ was downregulated in the anti-CD80/86 injection group compared with that in the phosphate-buffered saline (PBS) group (n = 3, *N.S* no significant difference, **P = 0.005). IL-1β was downregulated in the anti-CD80/86 injection group compared with that in the PBS group (n = 3, **P = 0.002). IL-2 was downregulated in anti-CD80/86 injection group compared with that in the PBS group (n = 3, **P = 0.003). TNF-α was downregulated in the anti-CD80/86 injection group compared with that in the PBS group (n = 3, ***P < 0.001). There was no difference in IL-10 between the anti-CD80/86 injection group and the PBS group (n = 3, N.S, *P* = 0.111). TGF-β1 was upregulated in the anti-CD80/86 injection group compared with that in the PBS group (n = 3, **P = 0.003). An unpaired *t-*test was applied for all statistical analyses.

Two weeks after transplantation, corneal grafts were collected from anti-CD80/86 antibody-injected mice and PBS-injected mice. The relative mRNA expression of IFN-γ, IL1-β, IL-2, and TNF-α in the grafts was downregulated in anti-CD80/86 injection group compared to PBS injection group (Fig. 5; n = 3; *P* = 0.005, *P* = 0.002, *P* = 0.003, and *P* < 0.001, respectively). The mRNA expression of TGF-β was highly upregulated in the anti-CD80/86 injection group (n = 3; *P* = 0.003). The mRNA expression of IL-10 was not significantly different between the groups (n = 3; *P* = 0.111). There was no difference in the mRNA expression of IL-12A and IL-12B between the groups (Supplementary Fig. S3).

### Differentially expressed genes (DEGs) in the anti-CD80/86 antibody-injected graft cornea

**Figure 6:**
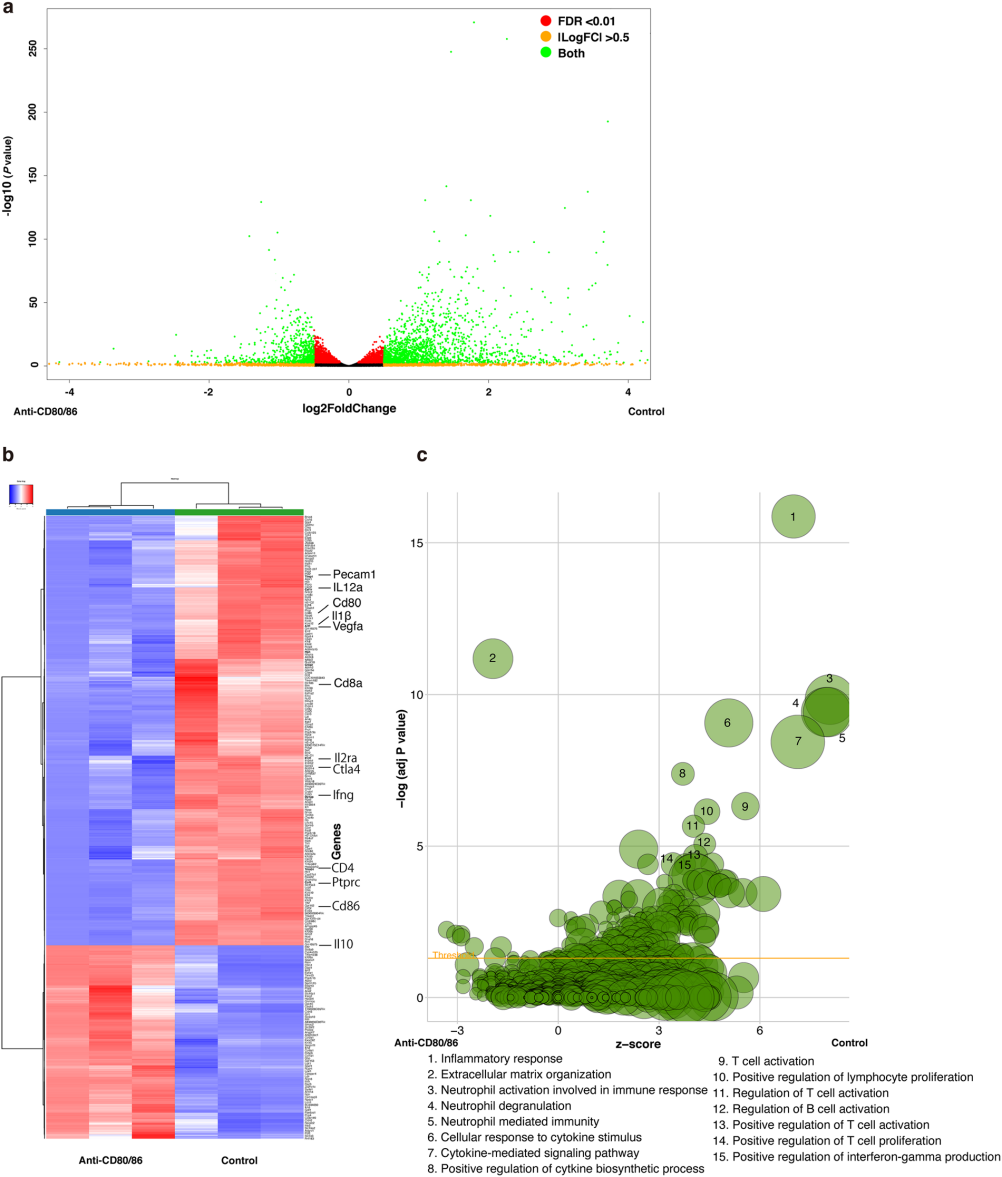
Differentially expressed genes and GO terms of differentially expressed genes between anti-CD80/86 injection and control corneal grafts. (**a**) Volcano plot of differentially expressed genes (DEGs) of corneal grafts between the anti-CD80/86 and phosphate-buffered saline (PBS) injection groups (control). The gene sets with a false discovery rate (FDR) of less than 0.01 were used as a threshold to determine the significance of DEGs. Green dots represent DEGs, and red dots indicate transcripts that did not significantly change between the anti-CD80/86 and PBS injection groups (control). (**b**) Hierarchical clustering of DEGs between the anti-CD80/86 injection group and the PBS injection group (control). The X axis represents the two compared samples (anti-CD80/86 injection and PBS injection group). The Y axis represents DEGs. The color (from blue to red) represents gene expression intensity from low to high. (**c**) The bubble plot of Gene Ontology terms. The z-score is assigned to the X axis and the negative logarithm of the *P* value to the Y axis, as observed in the bar plot (the higher the more significant). The area of the displayed circles is proportional to the number of genes assigned to the terms. Only the immunologically enriched GO terms’ labels are displayed.

To analyze the effect of anti-CD80/86 antibodies on corneal grafts, RNA-sequencing (RNA-Seq) (Supplementary Table S1 shows the RNA-Seq read statistics) and gene set enrichment analysis (GSEA) were performed. Figure 6a shows the volcano plot of DEGs in the corneal grafts between the anti-CD80/86-injected and control (PBS injection) groups. Among them, the expression of 672 and 1492 genes was significantly higher in the anti-CD80/86 and control groups, respectively. Figure 6b shows the hierarchical clustering of the identified DEGs between the anti-CD80/86 injection and control groups. In particular, the expression of *Pecam1* (Cd31), *Il12a*, *cd80*, *Il1β*, vascular endothelial growth factor-a (*Vegfa*), *Cd8a*, *Il2ra*, *Ctla4*, *Ifng*, *Cd4*, *Ptprc* (CD45), *Cd86*, and *Il10* was significantly lower in the anti-CD80/86-injected graft cornea group than in the control group. The Gene Ontology (GO) term enrichment analysis results revealed the significantly enriched biological processes in the anti-CD80/86 injection (Supplementary Table S2) and control groups (Supplementary Table S3). Figure 6c shows a bubble plot of the identified immunological-related GO enrichment terms.

**Figure 7:**
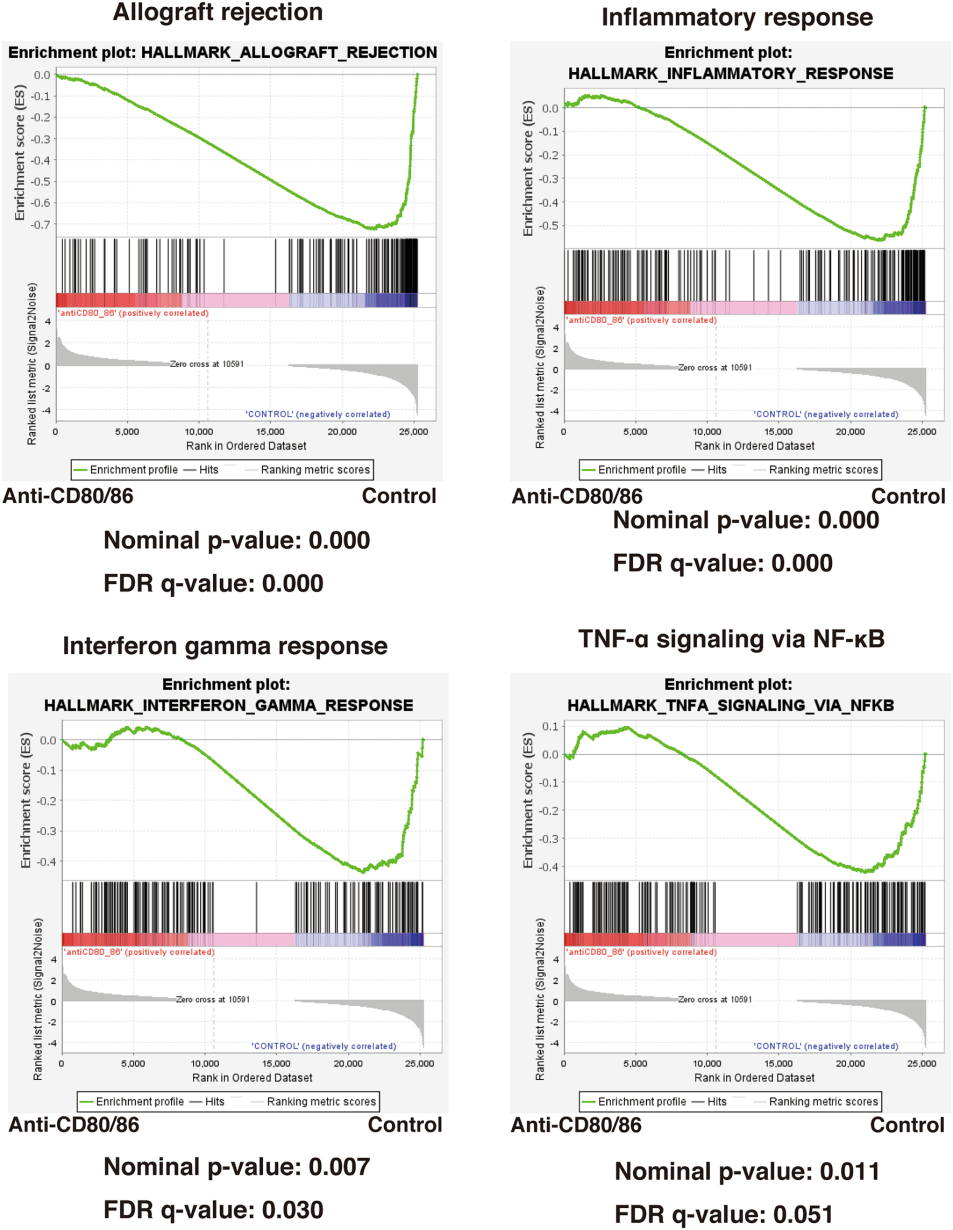
Gene set enrichment analysis (GSEA) of signaling pathways in high-risk corneal grafts between the anti-CD80/86 antibody injection and phosphate-buffered saline injection groups. GSEA querying hallmark genes (h.all.v6.0.symbols.gmt) depicting significant enrichment of Allograft Rejection genes (HALLMARK_ALLOGRAFT_REJECTION), Inflammatory response genes (HALLMARK_INFLAMMATORY_GENES), Interferon gamma response genes (HALLMARK_INTERFERON GAMMA RESPONSE), and TNF-α signaling via NFKB (HALLMARK_TNFA_SIGNALING_VIA_NFKB). *FDR* false discovery rate.

We compared the datasets for high-risk corneal grafts between the anti-CD80/86 antibody injection and control groups using the GSEA to identify the activated signaling pathways. Figure 7 shows the GSEA querying hallmark genes (h.all.v6.0.symbols.gmt), showing significant enrichment of Allograft Rejection genes, Inflammatory response genes, Interferon gamma response genes, and TNF-α signaling via NF-κB.

## Discussion

In this study, we investigated the suppressive effect of anti-CD80/86 antibodies in the local inflammatory microenvironment in a high-risk corneal rejection model. Our results demonstrated that blocking CD80 and CD86 conferred immunosuppression and prolonged graft survival by inhibiting T-cell proliferation and pro-inflammatory cytokine production. Therefore, anti-CD80/86 antibodies may be useful for suppressing immunological rejection in high-risk cases of corneal transplantation.

The interaction of CD80/CD86 on APCs and CD28 on T lymphocytes is an essential co-stimulatory pathway^20^, which could provide essential signals for T-cell growth, survival, activation, and proliferation^13^. This study showed that the injection of anti-CD80/86 antibodies inhibited T-cell proliferation both in vitro and in vivo. The GO term enrichment results revealed significant enrichment of the biological process T-cell activation and proliferation in the control group compared with that in the anti-CD80/86 injection group. These results suggest that anti-CD80/86 antibodies inhibit immune and inflammatory reactions by inhibiting T-cell proliferation.

CD4^+^ T cell infiltration and interaction with APCs at the graft site could promote dendritic cell maturation and activate IFN-γ-producing T cells^21^. Higher levels of IFN-γ have been correlated with graft rejection^22^. Ju et al. reported that both CD4^+^ and CD8^+^ T cells produce IFN-γ during acute rejection of an allogeneic transplant^23^. This study showed decreased frequencies of IFN-γ-producing CD4^+^ following treatment with anti-CD80/86 antibodies in the allogeneic stimulation. Antibodies counteracted relative CD80/86 exposure to T cells and, conversely, the activated T cells produced less IFN-γ due to insufficient co-stimulation. Furthermore, the GO term enrichment analysis and GSEA analysis revealed significant enrichment of the biological process IFN-γ response in the control group. Therefore, anti-CD80/86 antibodies could suppress Th1 immune response by reducing IFN-γ responses.

IFN-γ, IL-1β, and TNF-α are pro-inflammatory cytokines produced during immune responses, whereas IL-2 drives T-cell growth and induces Treg differentiation^24–27^. IL-2 is mainly produced by activated CD4^+^, generally in synergy with the level of IFN-γ^28^. We observed a similar elevation of IFN-γ and IL-2 in the supernatant used for MLR assay, and a similar reduction was observed when cells were treated with anti-CD80/86 antibodies. This could be attributed to the comprehensive suppression of T-cell proliferation. IL-1β, a potent proinflammatory cytokine that is crucial for host defense responses^27,29,30^, is mainly secreted by monocytes and macrophages as a part of the innate immune system^31,32^. The level of TNF-α, primarily produced by M1 macrophages^33,34^, is elevated in the rejection of solid organ transplants^35^. The reduced IL-1β and TNF-α expression in this study, achieved using anti-CD80/86 injection, indicates that the blockade of CD80/86 also suppresses the innate immune response. Furthermore, the GO term enrichment analysis also revealed significant enrichment of the biological processes, inflammatory responses, cellular response to cytokine stimulus, and cytokine-mediated signaling pathway in the control group. In the GSEA analysis, inflammatory response and TNF-α signaling were identified as activated pathways. In summary, inflammatory cytokines and relative genes expression were suppressed by the treatment with anti-CD80/86 antibodies in this high-risk rejection model.

IL-10 is known for their anti-inflammatory properties, including the ability to inhibit activation and effector function of T cells, shifting T cells towards a tolerogenic phenotype^36,37^. In this study, anti-CD80/86 antibodies decreased the production of IL-10 in vitro in accordance with that findings of a previous study^38^. However, we observed no significant difference in IL-10 mRNA expression in the corneal graft in vivo. On the contrary, as a major IL produced by dendritic cells, IL-12 showed no significant differences in production in the MLR supernatant and mRNA expression in corneal grafts. This discrepancy may be due to the difference in conditions between in vitro and in vivo^39^. The in vitro MLR experiments merely justified the possible mechanism of direct pathway of APCs involved in allogeneic sensitization, but not the indirect pathway. In vivo, the mRNA expression of IL-12 in the grafts of the two groups was similar; this result is consistent with the findings of Torres et al.^40^. Torres et al. assessed IL-12p40 mRNA level in experimental corneal allografts by selectively eliminating dendritic cells, and only observed a transient increase on post-grafting day 3, but not at a later time (days 7–17). Meanwhile, the RNA-Seq results in this study showed a lower level of the DEG *Il12a* in the grafts with anti-CD80/86 injection compared to PBS injection. These results suggest that IL-12 may play a more complex role in the development of Th1 responses. In summary, in the presence of anti-CD80/86 antibodies, a comprehensive suppression was achieved, and this process combined with the reduction in the level of inflammatory cytokines, such as IFN-γ, IL-1β, TNF-α, IL-2, and IL-10.

This study had some limitations. First, anti-CD80/86 antibodies block the presentation of CD80/86, which inhibits the co-stimulation pathway for T-cell proliferation. However, it remains unclear how this blockade is associated with other mechanisms; for example, we could not clarify the roles and activities APCs in the rejection process, and how the direct and indirect pathways play their respective roles in allograft rejection as the study was based on the one-way MLR in vitro assay. Further studies are needed to investigate the influence on the CD28-CTLA-4 interaction of CD80/86 blockade^41^. Second, we only performed minimal antibody injections early after grafting because we considered the strongest corneal inflammation generally occurred in the early stage of grafting^42^. A longer injection in a previous study showed that very few CD86^+^ or CD80^+^ cells were observed in the cornea, cervical lymph node, and spleen from the mice treated with anti-CD80/CD86 antibodies^40^. However, their effects on APC migration were not fully investigated, and observations with longer injections should be considered. Third, in this study, we only performed graft mRNA analysis at 2 weeks after transplantation; however, analysis at other time points such as mid- or late-stages of transplantation is necessary.

Our study revealed that the inhibitory effects of anti-CD80/86 antibodies prolonged the survival of high-risk rejection corneal grafts. Blockade of the CD80/86 signaling pathway inhibits CD4^+^ T-cell proliferation, lowers pro-inflammatory cytokine production, and may preserve Treg suppressive function. Therefore, anti-CD80/86 antibodies may be useful for suppressing immunological rejection in high-risk rejection of corneal transplantation.

## Methods

### Animals and anesthesia

Six–eight-week-old BALB/c (H-2d) and C57BL/6 (H-2b) male mice (B6) were obtained from Sankyo Labo Service Corporation, Inc. (Tokyo, Japan). All animal experiments were approved by the Institutional Animal Care and Use Committee of the Juntendo University Graduate School of Medicine (Approval No. 2020231); all experiments were conducted in accordance with the Association for Research in Vision and Ophthalmology Statement for the Use of Animals in Ophthalmic and Vision Research, and carried out in compliance with the ARRIVE guidelines.

Intraperitoneal anesthetic (ketamine-xylazine solution; 120 mg/kg body weight and 20 mg/kg body weight) was administered to the mice to induce general anesthesia.

### Generation of anti-CD80 and -CD86 monoclonal antibody (mAb)

Anti-mouse CD80 (RM80, rat IgG2a) and anti-CD86 (GL1, rat IgG2b) mAbs were generated as previously described^43^.

### Carboxyfluorescein diacetate succinimidyl ester-labeling

RPMI-1640 medium (Lonza, Basel, Switzerland) supplemented with 1 mg/mL streptomycin sulfate (Meiji Seika, Tokyo, Japan) was used as the complete medium. BALB/c mouse lymphocytes (1 × 10^7^ cells/mL) were incubated with carboxyfluorescein diacetate succinimidyl ester (CFSE) (eBioscience, San Diego, CA, USA) at a final concentration of 1 μM in pre-warmed PBS for 10 min at 20–22 °C in the dark. The labeling was stopped by adding complete media (5 mL), followed by incubation on ice for 5 min. The cells were then washed thrice with the complete medium. Finally, the CFSE-labeled cells were counted with an automated cell counter (Bio-Rad, Hercules, CA, USA) and immediately used in the MLR assay.

### MLR assay

In vitro, lymphocytes procured from BALB/c mice were selected as responder cells, and 30-Gy irradiated B6 mouse splenocytes were used as stimulators. The responder and stimulator cells were mixed at a concentration of 1 × 10^6^ cells/mL^44^. Proliferation was measured using the 5-bromo-2ʹ-deoxyuridine (BrdU) incorporation assay (EMD Millipore, Billerica, MA, USA) with 2 μg/mL anti-CD3 antibody CD3e (Clone: 145-2c11; BioLegend Inc., San Diego, CA, USA). The cells were co-cultured with and without anti-CD80 and anti-CD86 antibodies (10 μg/mL, respectively) and incubated for 4 days at 37 °C. On day 4, the culture supernatants were collected for an ELISA.

### ELISA

Supernatants were collected for ELISA on day 4 of the MLR assay. ELISA kits for mouse IFN-γ (DY485), IL-2 (DY402-05), IL-10 (DY417-05), TGF-β1 (DY1679-05) (R&D Systems, Inc., Minneapolis, MN, USA), and IL-12 (433604) (BioLegend, San Diego, CA, USA) were used according to the manufacturers’ instructions.

### High-risk allogeneic corneal transplantation and graft survival

Inflamed, neovascularized, “high-risk” recipient beds were created^7,8,45^ by placing three intrastromal sutures into the central cornea 14 days before corneal transplantation. As described previously^7–9,46,47^, C57BL/6 corneas were grafted onto BALB/c host beds. The central cornea was excised from donor C57BL/6 mice. The graft bed was prepared by excising the central cornea from BALB/c mice. The donor button was then placed onto the recipient bed and secured with eight interrupted 11-0 nylon sutures, which were removed 7 days after the surgery. Ofloxacin ophthalmic ointment 0.3% (Pharmaceutical Co., Ltd., Osaka, Japan) was applied on the graft cornea immediately after corneal transplantation.

Graft neovascularization score, opacity score, and survival rate were evaluated using a slit lamp biomicroscope^7,45,47^. We used a standardized scoring system to calculate the neovascularization (range 0–8) and opacity (range 0–5+) scores. Corneas with an opacity score of 2 + for two consecutive examinations were considered rejected. Scoring was performed every 7 days for 8 weeks.

### Intraperitoneal injection of anti-CD80/86 antibodies

The recipient mice were administered anti-CD80 and anti-CD86 mAb (50 μg), reconstituted in normal saline (100 μL). The injection was administered after grafting on days 0, 1, and 2 (n = 6, each). The control mice were injected with PBS.

### Flow cytometry

A single-cell suspension of co-culture cells and grafted corneas was prepared as previously described^7,8,48^. To avoid non-specific staining, the cells were blocked with an anti-FcR-blocking antibody (eBioscience, San Diego, CA, USA). The isolated cells were stained with the following antibodies: FITC-anti-CD4 (RM4-5), and anti-IFN-γ (XMG1.2) antibodies (BioLegend, San Diego, CA, USA). The stained cells were examined using flow cytometry and the data were analyzed using FlowJo software X 10.5.3 (FlowJo LLC, Ashland, OR, USA).

### Corneal RNA extraction and RT-PCR

Two weeks after transplantation, the corneal grafts were excised and immediately submerged in an RNAlater solution (Ambion, Austin, TX, USA). The total RNA from five corneas was isolated for each group using a NucleoSpin RNA isolation kit (Macherey–Nagel GmbH, Duren, Germany). Next, cDNA was prepared using the ReverTra Ace qPCR RT kit (Toyobo, Osaka, Japan). Thereafter, RT-qPCR was performed, with all reactions performed thrice. The results were analyzed using the 2^−ΔΔCt^ method, and *Gapdh* was used as an internal control. Specific primer sets were used for *Ifnγ, Tgfb1, Il1b, Il2, Il10, Tnfa*, *Il12a, Il12b*, and *Gapdh* (Supplementary Table S4).

### RNA-seq library preparation and sequencing

RNA library preparation and sequencing were conducted at GENEWIZ, LLC (South Plainfield, NJ, USA). The cDNA sequencing libraries were prepared using NEBNext Poly(A) mRNA Magnetic Isolation Module and NEBNext Ultra II RNA Library Prep Kit for Illumina, following the manufacturer’s recommendations (NEB, Ipswich, MA, USA). The sequencing libraries were validated on the Agilent TapeStation System (Agilent Technologies, Palo Alto, CA, USA), and quantified using a Qubit 2.0 Fluorometer (Invitrogen, Carlsbad, CA, USA) and quantitative PCR (Applied Biosystems, Carlsbad, CA, USA). The sequencing libraries were sequenced as 2 × 150 paired-end (PE) using the Illumina NovaSeq Sequencing System. After sequencing, Trimmomatic (version 0.39) was used to remove adapters and filter the raw reads with < 35 bases, as well as the leading and trailing bases with quality < 20. The filtered reads were mapped to the sequence of the mouse genome (GRCm38) using HISAT2 (v2.1.0). Raw counts for each gene were obtained using featureCounts software included with the Subread package with the default option (-p -t exon). RUVSeq (version 1.18.0) was used for further normalization to account for sample variations.

### Identification and analysis of differentially expressed genes

DEGs were identified using DESeq2 (version 1.24.0) analysis with a threshold padj < 0.01 and abs (Log2 FC) > 0.5. Gene set enrichment analysis of the RNA-Seq data was performed using GSEA software (version 4.0.3, https://www.gsea-msigdb.org/gsea/index.jsp) and MSigDB v 7.1, focusing on H hallmark gene sets. Log normalized sample counts, following GSEA parameters, were used: norm = meandiv, nperm (number of permutations) = 1000, and permute (permutation type) was set to “permutation on gene set” because of the low number of samples employed. Gene sets with a false discovery rate < 0.25 were considered significant. The raw and processed RNA-Seq data were deposited in the NCBI GEO database (accession number: GSE181893).

### Statistical analysis

Mann–Whitney *U* test was used to compare means. Data of more than two groups were analyzed using the one-way or two-way analysis of variance (ANOVA) with post-hoc Bonferroni’s multiple comparison test. Kaplan–Meier analysis with the log-rank test was used to evaluate graft survival after transplantation. Data are presented as mean or median ± standard error of the mean and considered statistically significant at *P* < 0.05. All statistical calculations were performed using Prism Version 8.4.3 software (GraphPad, La Jolla, CA, USA).

## Supplementary Information

Supplementary Figures.

Supplementary Table S1.

Supplementary Table S2.

Supplementary Table S3.

Supplementary Table S4.

## Data Availability

All datasets generated during or analyzed during this study are included in this published article.
