# Supplementary material for: Anti-CD80/86 antibodies inhibit inflammatory reaction and improve graft survival in a high-risk murine corneal transplantation rejection model
Source: Sci Rep. 2022 Mar 22;12:4853. doi: 10.1038/s41598-022-08949-9 (PMC8941080; doi:10.1038/s41598-022-08949-9)
Supplement: Supplementary file 1 — Supplementary Figures. [file 41598_2022_8949_MOESM1_ESM.pdf]

Supplementary Information

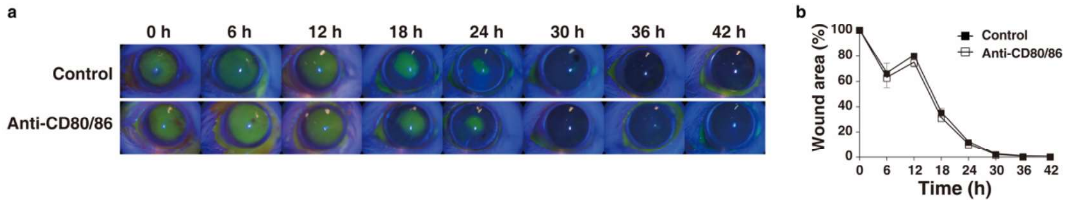

Supplementary Figure S1. Corneal epithelial wound healing assay following anti-CD80/86 antibody injection. Six eight-week-old BALB/c (H-2d) male mice were anesthetized and used for the corneal epithelial wound healing assay. A murine corneal wound model was generated by manually creating a 2 mm-diameter wound at the center of the cornea of the right eye using a 2-mm trephine. Epithelial defects were made using the sharp point of an Oonishi knife (Toyohara Ophthalmic Instruments Limited Co., Tokyo, Japan). The effects of anti-CD80/86 injection on the corneal wound healing were compared with those of a phosphate-buffered saline (PBS) injection in the murine corneal wound model (n = 5 per group). Each injection was administrated immediately after the surgery. Each corneal wound was stained with 0.5% fluorescein and monitored every 6 h after surgery using a slit-lamp microscope until the wound recovered completely. The area of the corneal epithelial defect was calculated using ImageJ software; version 1.53a. (a) Images representing the corneal epithelial wound healing assay. No significant differences were observed among the anti-CD80/80 antibodies or phosphate-buffered saline (PBS) injection groups (two-way ANOVA, n = 5/group, median wound closure time; anti-CD80/86 injection 42 [0–42] h *vs* PBS injection 42 [0–42] h, $P = 0.131$) in the corneal epithelial wound healing areas.

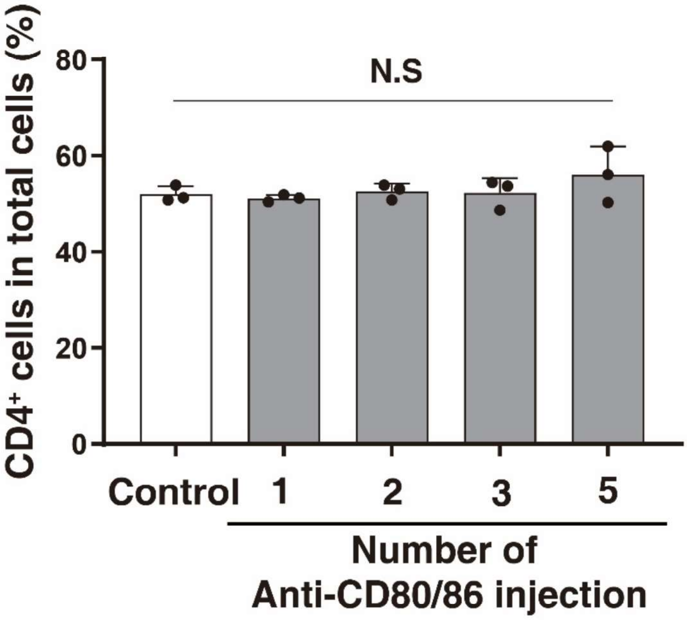

Supplementary Figure S2. CD4$^+$ T cell proportion in draining lymph nodes cells after anti-CD80/86 antibody injection. The anti-CD80/86 antibody was administered on days 1, 2, 3, and 5, once a day. On day 7, the corneas and ipsilateral draining lymph nodes were harvested, and single-cell suspensions were prepared as described in the methods section. To avoid non-specific staining, cells were blocked with an anti-FcR blocking antibody (eBioscience, San Diego, CA, USA). The isolated cells were stained with the respective antibodies: anti-CD45 PE (30-F11, eBioscience) and anti-CD4 FITC (GK1.5, eBioscience). The stained cells were examined using LSR Fortessa (BD Biosciences, Franklin Lakes, NJ, USA). The data was analyzed using FlowJo software X 10.5.3. (FlowJo LLC, Ashland, OR, USA; purchased from https://www.flowjo.com). No differences were observed in the proportion of CD4$^+$ T cells among all the draining lymph node cells of the anti-CD80/86 injection and control groups (one-way ANOVA, n = 3/group, P = 0.406).

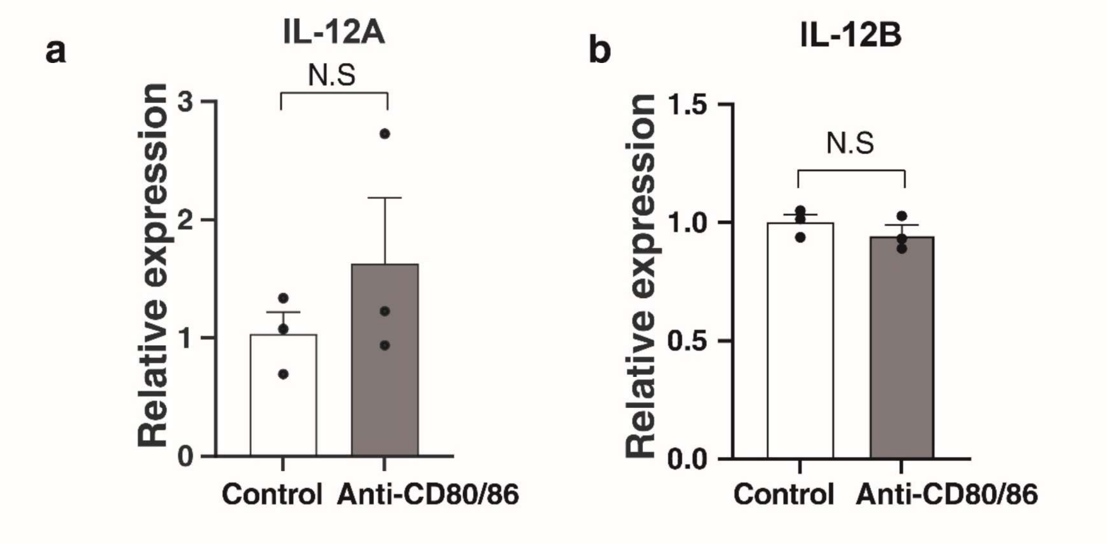

Supplementary Figure S3. Cytokine mRNA expression. in corneal grafts after anti-CD80/86 antibody injection. Two weeks after transplantation, the corneal grafts were excised and immediately submerged in an RNAlater solution (Ambion, Austin, TX, USA). Total RNA was isolated from five corneas in each group using a NucleoSpin RNA isolation kit (Macherey-Nagel GmbH, Duren, Germany). Subsequently, cDNA was prepared using the ReverTra Ace qPCR RT kit (Toyobo, Osaka, Japan). Thereafter, RT-qPCR was performed; all reactions were performed in triplicate. The results were analyzed using the $2^{-\Delta\Delta Ct}$ method, and *Gapdh* was used as an internal control (N.S: no significant difference, IL-12A: n = 3, *P* = 0.367; IL-12B: n = 3, *P* = 0387).
