# Supplementary material for: Anti-CD80/86 antibodies inhibit inflammatory reaction and improve graft survival in a high-risk murine corneal transplantation rejection model
Source: Sci Rep. 2022 Mar 22;12:4853. doi: 10.1038/s41598-022-08949-9 (PMC8941080; doi:10.1038/s41598-022-08949-9)
Supplement: Supplementary file 2 — Supplementary Table S1. [file 41598_2022_8949_MOESM2_ESM.docx]

**Supplementary Table S1. The RNA-seq read statistics**

| Group | Anti-CD80/86 injection | | | Phosphate-buffered saline injection (Control) | | |
| --- | --- | --- | --- | --- | --- | --- |
| Sample number | 1 | 2 | 3 | 1 | 2 | 3 |
| Sequenced Reads | 38,189,292 | 42,795,240 | 42,588,736 | 40,408,510 | 41,276,809 | 39,807,679 |
| Mapped Reads | 34,706,533 | 38,930,254 | 38,430,554 | 36,252,471 | 36,981,303 | 35,719,832 |
| Mapping Rate | 97.29% | 97.24% | 97.16% | 96.78% | 96.68% | 96.84% |
| Expression Profiling Efficiency | 0.847 | 0.847 | 0.852 | 0.83 | 0.828 | 0.835 |
| High Quality Rate | 0.777 | 0.783 | 0.786 | 0.784 | 0.789 | 0.774 |
| Exonic Rate | 0.871 | 0.871 | 0.877 | 0.857 | 0.856 | 0.863 |
| Intronic Rate | 0.075 | 0.076 | 0.07 | 0.085 | 0.085 | 0.08 |
| Intergenic Rate | 0.023 | 0.022 | 0.021 | 0.023 | 0.024 | 0.023 |
| Intragenic Rate | 0.946 | 0.947 | 0.947 | 0.943 | 0.941 | 0.943 |
| rRNA Rate | 0.003 | 0.002 | 0.002 | 0.002 | 0.002 | 0.002 |
| Read Length | 150 | 150 | 150 | 150 | 150 | 150 |
| Genes Detected | 18,200 | 18,272 | 17,917 | 18,061 | 18,301 | 18,186 |
