# Supplementary material for: Anti-CD80/86 antibodies inhibit inflammatory reaction and improve graft survival in a high-risk murine corneal transplantation rejection model
Source: Sci Rep. 2022 Mar 22;12:4853. doi: 10.1038/s41598-022-08949-9 (PMC8941080; doi:10.1038/s41598-022-08949-9)
Supplement: Supplementary file 3 — Supplementary Table S2. [file 41598_2022_8949_MOESM3_ESM.docx]

**Supplementary Table S2. Gene Ontology (GO) enrichment results in the anti-CD80/86 injection group**

| Term | Overlap | *P* value | Adjusted *P* value |
| --- | --- | --- | --- |
| Extracellular matrix organization (GO:0030198) | 42/229 | 1.70E-19 | 4.07E-16 |
| Collagen fibril organization (GO:0030199) | 44,529 | 1.13E-09 | 1.36E-06 |
| Sterol biosynthetic process (GO:0016126) | 12/40 | 4.43E-09 | 3.54E-06 |
| Cholesterol biosynthetic process (GO:0006695) | 11/35 | 1.14E-08 | 6.81E-06 |
| Secondary alcohol biosynthetic process (GO:1902653) | 11/36 | 1.59E-08 | 7.61E-06 |
| Sulfur compound biosynthetic process (GO:0044272) | 18/122 | 1.36E-07 | 5.45E-05 |
| Protein complex subunit organization (GO:0071822) | 11/45 | 2.04E-07 | 6.97E-05 |
| Cholesterol metabolic process (GO:0008203) | 12/68 | 2.44E-06 | 7.32E-04 |
| Modulation of chemical synaptic transmission (GO:0050804) | 13/82 | 3.28E-06 | 8.73E-04 |
| Nervous system development (GO:0007399) | 34/455 | 1.29E-05 | 2.84E-03 |
| Regulation of alcohol biosynthetic process (GO:1902930) | 8/34 | 1.30E-05 | 2.84E-03 |
| Glycosaminoglycan biosynthetic process (GO:0006024) | 13/99 | 2.69E-05 | 5.38E-03 |
| Regulation of cholesterol biosynthetic process (GO:0045540) | 8/40 | 4.62E-05 | 8.53E-03 |
| Regulation of cholesterol metabolic process (GO:0090181) | 8/41 | 5.58E-05 | 9.56E-03 |
| Regulation of steroid biosynthetic process (GO:0050810) | 8/44 | 9.47E-05 | 1.46E-02 |
| Regulation of endothelial cell migration (GO:0010594) | 10/69 | 9.75E-05 | 1.46E-02 |
| Modulation of excitatory postsynaptic potential (GO:0098815) | 44372 | 1.44E-04 | 2.04E-02 |
| Peptide metabolic process (GO:0006518) | 12/104 | 1.93E-04 | 2.35E-02 |
| Glutathione metabolic process (GO:0006749) | 8/49 | 2.08E-04 | 2.35E-02 |
| Dorsal/ventral axis specification (GO:0009950) | 44296 | 2.26E-04 | 2.35E-02 |
| Negative regulation of inclusion body assembly (GO:0090084) | 44296 | 2.26E-04 | 2.35E-02 |
| Positive regulation of cellular component organization (GO:0051130) | 12/106 | 2.31E-04 | 2.35E-02 |
| Regulation of ossification (GO:0030278) | 7/38 | 2.38E-04 | 2.35E-02 |
| Kidney development (GO:0001822) | 9/63 | 2.42E-04 | 2.35E-02 |
| Negative regulation of signal transduction (GO:0009968) | 22/283 | 2.45E-04 | 2.35E-02 |
| Negative regulation of cell proliferation (GO:0008285) | 26/363 | 2.58E-04 | 2.37E-02 |
| Visual perception (GO:0007601) | 10/78 | 2.75E-04 | 2.37E-02 |
| Positive regulation of ossification (GO:0045778) | 8/52 | 3.17E-04 | 2.37E-02 |
| Regulation of angiogenesis (GO:0045765) | 16/177 | 3.24E-04 | 2.37E-02 |
