# Supplementary material for: Anti-CD80/86 antibodies inhibit inflammatory reaction and improve graft survival in a high-risk murine corneal transplantation rejection model
Source: Sci Rep. 2022 Mar 22;12:4853. doi: 10.1038/s41598-022-08949-9 (PMC8941080; doi:10.1038/s41598-022-08949-9)
Supplement: Supplementary file 4 — Supplementary Table S3. [file 41598_2022_8949_MOESM4_ESM.docx]

**Supplementary Table S3. Gene Ontology (GO) enrichment results in the PBS injection (control) group**

| Term | Overlap | *P* value | Adjusted *P* value |
| --- | --- | --- | --- |
| Extracellular matrix organization (GO:0030198) | 42/229 | 1.70E-19 | 4.07E-16 |
| Collagen fibril organization (GO:0030199) | 44,529 | 1.13E-09 | 1.36E-06 |
| Sterol biosynthetic process (GO:0016126) | 12/40 | 4.43E-09 | 3.54E-06 |
| Cholesterol biosynthetic process (GO:0006695) | 11/35 | 1.14E-08 | 6.81E-06 |
| Secondary alcohol biosynthetic process (GO:1902653) | 11/36 | 1.59E-08 | 7.61E-06 |
| Sulfur compound biosynthetic process (GO:0044272) | 18/122 | 1.36E-07 | 5.45E-05 |
| Protein complex subunit organization (GO:0071822) | 11/45 | 2.04E-07 | 6.97E-05 |
| Cholesterol metabolic process (GO:0008203) | 12/68 | 2.44E-06 | 7.32E-04 |
| Modulation of chemical synaptic transmission (GO:0050804) | 13/82 | 3.28E-06 | 8.73E-04 |
| Nervous system development (GO:0007399) | 34/455 | 1.29E-05 | 2.84E-03 |
| Regulation of alcohol biosynthetic process (GO:1902930) | 8/34 | 1.30E-05 | 2.84E-03 |
| Glycosaminoglycan biosynthetic process (GO:0006024) | 13/99 | 2.69E-05 | 5.38E-03 |
| Regulation of cholesterol biosynthetic process (GO:0045540) | 8/40 | 4.62E-05 | 8.53E-03 |
| Regulation of cholesterol metabolic process (GO:0090181) | 8/41 | 5.58E-05 | 9.56E-03 |
| Regulation of steroid biosynthetic process (GO:0050810) | 8/44 | 9.47E-05 | 1.46E-02 |
| Regulation of endothelial cell migration (GO:0010594) | 10/69 | 9.75E-05 | 1.46E-02 |
| Modulation of excitatory postsynaptic potential (GO:0098815) | 44372 | 1.44E-04 | 2.04E-02 |
| Peptide metabolic process (GO:0006518) | 12/104 | 1.93E-04 | 2.35E-02 |
| Glutathione metabolic process (GO:0006749) | 8/49 | 2.08E-04 | 2.35E-02 |
| Dorsal/ventral axis specification (GO:0009950) | 44296 | 2.26E-04 | 2.35E-02 |
| Negative regulation of inclusion body assembly (GO:0090084) | 44296 | 2.26E-04 | 2.35E-02 |
| Positive regulation of cellular component organization (GO:0051130) | 12/106 | 2.31E-04 | 2.35E-02 |
| Regulation of ossification (GO:0030278) | 7/38 | 2.38E-04 | 2.35E-02 |
| Kidney development (GO:0001822) | 9/63 | 2.42E-04 | 2.35E-02 |
| Negative regulation of signal transduction (GO:0009968) | 22/283 | 2.45E-04 | 2.35E-02 |
| Negative regulation of cell proliferation (GO:0008285) | 26/363 | 2.58E-04 | 2.37E-02 |
| Visual perception (GO:0007601) | 10/78 | 2.75E-04 | 2.37E-02 |
| Positive regulation of ossification (GO:0045778) | 8/52 | 3.17E-04 | 2.37E-02 |
| Regulation of angiogenesis (GO:0045765) | 16/177 | 3.24E-04 | 2.37E-02 |

| Term | Overlap | *P* value | Adjusted *P* value |
| --- | --- | --- | --- |
| Inflammatory response (GO:0006954) | 72/252 | 4.39E-24 | 1.56E-20 |
| Neutrophil activation involved in immune response (GO:0002283) | 96/483 | 3.97E-19 | 7.06E-16 |
| Neutrophil mediated immunity (GO:0002446) | 95/487 | 2.29E-18 | 2.04E-15 |
| Neutrophil degranulation (GO:0043312) | 94/479 | 2.30E-18 | 2.04E-15 |
| Cytokine-mediated signaling pathway (GO:0019221) | 103/633 | 3.15E-14 | 2.24E-11 |
| T cell activation (GO:0042110) | 31/88 | 7.18E-14 | 4.25E-11 |
| Positive regulation of cytokine biosynthetic process (GO:0042108) | 19/40 | 9.83E-12 | 5.00E-09 |
| Positive regulation of lymphocyte proliferation (GO:0050671) | 25/71 | 1.88E-11 | 8.37E-09 |
| Cellular response to cytokine stimulus (GO:0071345) | 76/456 | 2.48E-11 | 9.80E-09 |
| Regulation of B cell proliferation (GO:0030888) | 19/43 | 4.83E-11 | 1.72E-08 |
| Regulation of T cell activation (GO:0050863) | 19/44 | 7.90E-11 | 2.55E-08 |
| Cellular defense response (GO:0006968) | 21/56 | 1.97E-10 | 5.84E-08 |
| Mitotic sister chromatid segregation (GO:0000070) | 25/82 | 6.41E-10 | 1.75E-07 |
| Muscle contraction (GO:0006936) | 33/137 | 1.23E-09 | 2.97E-07 |
| Regulation of immune response (GO:0050776) | 48/251 | 1.25E-09 | 2.97E-07 |
| B cell activation (GO:0042113) | 23/73 | 1.50E-09 | 3.14E-07 |
| Lymphocyte differentiation (GO:0030098) | 23/73 | 1.50E-09 | 3.14E-07 |
| Response to molecule of bacterial origin (GO:0002237) | 27/98 | 1.66E-09 | 3.28E-07 |
| Positive regulation of T cell activation (GO:0050870) | 22/68 | 1.93E-09 | 3.62E-07 |
| Muscle filament sliding (GO:0030049) | 16/38 | 3.91E-09 | 6.59E-07 |
| Actin-myosin filament sliding (GO:0033275) | 16/38 | 3.91E-09 | 6.59E-07 |
| Positive regulation of interferon-gamma production (GO:0032729) | 17/43 | 4.08E-09 | 6.59E-07 |
| Response to lipopolysaccharide (GO:0032496) | 34/155 | 9.17E-09 | 1.42E-06 |
| Dendritic cell chemotaxis (GO:0002407) | 44485 | 2.74E-08 | 4.05E-06 |
| Positive regulation of cytosolic calcium ion concentration (GO:0007204) | 30/133 | 3.45E-08 | 4.91E-06 |
| Enzyme linked receptor protein signaling pathway (GO:0007167) | 28/120 | 4.53E-08 | 6.19E-06 |
| Positive regulation of T cell proliferation (GO:0042102) | 19/61 | 4.88E-08 | 6.43E-06 |
| Negative regulation of T cell activation (GO:0050868) | 14/34 | 5.22E-08 | 6.63E-06 |
| Sarcomere organization (GO:0045214) | 13/31 | 1.21E-07 | 1.49E-05 |
| Negative regulation of lymphocyte activation (GO:0051250) | 10/19 | 2.56E-07 | 3.04E-05 |
