# Supplementary material for: Anti-CD80/86 antibodies inhibit inflammatory reaction and improve graft survival in a high-risk murine corneal transplantation rejection model
Source: Sci Rep. 2022 Mar 22;12:4853. doi: 10.1038/s41598-022-08949-9 (PMC8941080; doi:10.1038/s41598-022-08949-9)
Supplement: Supplementary file 5 — Supplementary Table S4. [file 41598_2022_8949_MOESM5_ESM.docx]

**Supplementary Table S4. Primers sets for real-time PCR.**

|  |  |  |
| --- | --- | --- |
| **Gene** | **Forward (5′–3′)** | **Reverse (5′–3′)** |
| *Gapdh* | AAGGGCTCATGACCACAGTC | GGATGACCTTGCCCACAG |
| *Ifnγ* | CGGCACAGTCATTGAAAGCC | TGTCACCATCCTTTTGCCAGT |
| *Tnfa* | AGCCCACGTCGTAGCAAAC | TTTGAGATCCATGCCGTTGG |
| *Tgfb1* | AGACAAGAGCTCACCCACTGTC | AGAGCGAGGCCATCAGTCTATC |
| *Il10* | GGCGCTGTCATCGATTTCT | TGGCCTTGTAGACACCTTGG |
| *Il1b* | TGCCACCTTTTGACAGTGATG | ATGTGCTGCTGCGAGATTTG |
| *Il2* | GCGGCATGTTCTGGATTTGA | TGTGTTGTCAGAGCCCTTTAGTT |
| *Il12a* | TCCCTTGGATCTGAGCTGGA | TGTTGGAACGCTGACCATAGA |
| *Il12b* | TGGGAGTACCCTGACTCCTG | AGGAACGCACCTTTCTGGTT |
